# Hydrolysis of Selected Tropical Plant Wastes Catalyzed by a Magnetic Carbonaceous Acid with Microwave

**DOI:** 10.1038/srep17538

**Published:** 2015-12-09

**Authors:** Tong-Chao Su, Zhen Fang, Fan Zhang, Jia Luo, Xing-Kang Li

**Affiliations:** 1 University of Science and Technology of China, School of Life Science, 443 Huangshan Road, Hefei, Anhui Province, 230027, China; 2 Chinese Academy of Sciences, Biomass Group, Key Laboratory of Tropical Plant Resources and Sustainable Use, Xishuangbanna Tropical Botanical Garden, 88 Xuefulu, Kunming, Yunnan Province 650223, China; 3 University of Chinese Academy of Sciences, 19A Yuquan Road, Beijing 100049, China

## Abstract

In this study, magnetic carbonaceous acids were synthesized by pyrolysis of the homogeneous mixtures of glucose and magnetic Fe_3_O_4_ nanoparticles, and subsequent sulfonation. The synthesis conditions were optimized to obtain a catalyst with both high acid density (0.75 mmol g^−1^) and strong magnetism [magnetic saturation, Ms = 19.5 Am^2^ kg^−1^]. The screened catalyst (C-SO_3_H/Fe_3_O_4_) was used to hydrolyze ball-milled cellulose in a microwave reactor with total reducing sugar (TRS) yield of 25.3% under the best conditions at 190 °C for 3.5 h. It was cycled for at least seven times with high catalyst recovery rate (92.8%), acid density (0.63 mmol g^−1^) and magnetism (Ms = 12.9 Am^2^ kg^−1^), as well as high TRS yield (20.1%) from the hydrolysis of ball-milled cellulose. The catalyst was further successfully tested for the hydrolysis of tropical biomass with high TRS and glucose yields of 79.8% and 58.3% for bagasse, 47.2% and 35.6% for *Jatropha* hulls, as well as 54.4% and 35.8% for *Plukenetia* hulls.

South China is located in subtropical and tropical regions that are suitable to grow energy or economic crops, such as sugarcane, *Jatropha curcas* L. and *Plukenetia volubilis* L. plants. As planned by National Development and Reform Commission of China (2015), sugar production in China will increase from 128.2 in 2013 to 180 million tons in 2020^1^. As biodiesel trees, the growing area of *Jatropha* has expanded (including natural forests) to about 2 × 10^5^ ha in China^2^. *Plukenetia* is regarded as a potential biodiesel and health food plant because of the high protein (33%) and oil (49%) contents in its seeds^3^, and is planned to extend its growing area by Yunnan provincial government. With the development of these tropical crops in Southern China, particularly in Yunnan, more biomass residues will be produced and it is important to volatilize them, such as conversion to sugars by hydrolysis.

A great deal of effort has been made for the hydrolysis of cellulose to glucose with enzymes, acids and supercritical water^4^,^5^. The hydrolysis of lignocelluloses in concentrated and diluted homogenous acids with high sugar yield has been practiced for many years under different conditions^6^. However, problems such as corrosion hazards, acid-waste and difficulties in separation are not solved effectively. Various solid materials, such as transition metal oxides^7^, ion-exchange resins^8^, zeolites^9^ and sulfonated carbons^10^,^11^ are used as environmentally friendly catalysts for biomass hydrolysis because of their reusability, less corrosiveness and non-toxicity. Sulfonated carbonaceous catalysts effectively catalyze the hydrolysis of cellulose by their acidic groups such as -SO_3_H, -COOH and -OH^12^. Furthermore, some solid catalysts are made with magnetic design for easier separation from product mixtures^13^,^14^. However, many magnetization methods are introduced by chemical reactions, such as precipitation and subsequent reduction of Fe^3+^ to Fe or Fe_3_O_4_^4^,^15^,^16^ that may cause pollution and high cost.

Moreover, many techniques are developed for biomass pretreatment to reduce the recalcitrance and crystallinity of cellulose, and increase its surface area for improving hydrolysis efficiency and selectivity. These methods include ball-milling^17^, liquid acid/base^18^,^19^, ionic liquids^20^ and microwave irradiation^21^ treatments. Microwave irradiation selectively heats reactants in molecular level and remarkably accelerates chemical reactions that differs from conventional heating manners such as thermal heating^22^.

In this study, magnetic sulfonated acid was synthesized from glucose and magnetized without chemical reactions by simply mixing magnetic Fe_3_O_4_ nanoparticles with aqueous glucose solution. The catalyst was screened under different conditions for optimization of glucose production from ball-milled cellulose, and further used for the hydrolysis of selected tropical biomass wastes assisted by microwave.

## Methods

### Materials

Nano-Fe_3_O_4_ (≥99.5%, 20 nm) was purchased from Aladdin Factory Co., Ltd. (Shanghai). H_2_SO_4_ (≥98.0%) and microcrystalline cellulose (powder) were purchased from Xilong Chemical Factory Co., Ltd. (Shantou, Guangdong). Standard agents glucose, xylose, arabinose, mannose, formic acid, acetic acid, lactic acid, levulinic acid, 5- hydroxymethylfurfural (HMF) and furfural (purity >99%) were from Sigma-Aldrich (Shanghai). Cellulose was ball-milled with ZrO_2_ balls (SHQM-0.4L, Chunlong Petroleum Instrument Co., Ltd., Lianyungang, Jiangsu) for 24 h before use. Bagasse was purchased from Dehong (Yunnan). *Jatropha* hulls were purchased from Yunnan Shenyu New Energy Co., Ltd. (Chuxiong, Yunnan). *Plukenetia* hulls were collected from Xishuangbanna Tropical Botanical Garden (Mengla, Yunnan). The raw materials were grounded in a pulverizer (9FC-15, Xudong machinery manufacturing Co., Ltd., Leshan, Sichuan) and dried at 45 °C until constant weight. All tropical biomass samples were sieved through 80 mesh for pretreatment and hydrolysis. Bagasse, *Jatropha* and *Plukenetia* hulls were also ball-milled for 24 h, water-extracted for 24 h, as well as water- and ethanol-extracted for 48 h at their boiling points in a Soxhlet extractor for hydrolysis.

### Catalyst preparation

For a typical procedure, nano-Fe_3_O_4_ (2 g) and glucose (12–18 g) were mixed with 100 mL water in a three-neck flask under vigorous stirring in an oil bath at 100 °C. After water was evaporated, the mixture was transferred into a tubular furnace (SGL-1100, Shanghai Daheng Optics and Fine Mechanics Co., Ltd.), heated to 650–750 °C at rate of 5–7 °C /min and pyrolyzed for 0.5–1.5 h under N_2_ flow (280 mL min^−1^). The carbonized solid C/Fe_3_O_4_ was sulfonated by 98% H_2_SO_4_ with the ratio of 1 g/20 mL in oil bath at 150 °C for 19–21 h. The sulfonated sample was washed repeatedly with distilled water at 200 °C for 3 h in a 50 mL high-pressure micro-autoclave (YZPR-50, Shanghai Yanzheng Experimental Instrument Co., Ltd.) until neutral solution was reached (and no SO_4_^2−^ was detected using CaCl_2_). The washed catalyst was dried in an oven (WFO-710, EYELA, Tokyo Rikakikai Co., Ltd.) at 105 °C for 24 h until constant weight, ground and sieved through 200 mesh for hydrolysis.

### Catalyst characterization

The morphology of catalyst was examined by scanning electron microscopy (SEM, ZEISS EVO LS10, Cambridge, UK). The crystalline structure was analyzed by X-ray diffraction (XRD, Rigaku Rotaflex RAD-C, Tokyo) equipped with a Cu Kα radiation source (40 kv and 200 mA). Fourier transform-infrared (FT-IR) measurement for catalysts and biomass was recorded on Nicolet *is*10 spectrometer with a spectral resolution of 4 cm^−1^ in the wavenumber range of 500–4000 cm^−1^. The magnetism of catalyst was measured by a vibrating sample magnetometer (VSM, 7407, Lake Shore Cryotronics, Inc. Westerville, OH). The element composition of catalyst was determined by a 2400 Series II CHNS/O Elemental Analyzer (PerkinElmer, Waltham, MA), and the acid density was calculated by sulfur content. Ammonia temperature programmed desorption (NH_3_-TPD, Chemisorption analyzer, Quantachrome Instruments, Boynton Beach, FL) and NaOH titration were also used to determine the acid density. In NH_3_-TPD analysis, catalyst (20–150 mg) was treated under NH_3_ flow (10% NH_3_ and 90% He) for absorption at 50 °C for 60 min after degassing, and heated to 400 °C under pure He with a heating rate of 5 °C/min for desorption. The background curve was obtained by treating catalyst under pure He instead of NH_3_ flow in the adsorption step. In NaOH titration analysis, catalyst sample (0.1 g) was mixed with NaOH solution (50 mL, 0.02 mol/L) and ultrasonicated for neutralizing the surface acidity of catalyst. The consumed base concentration was back-titrated by HCl solution (0.02 mol/L) with phenolphthalein as the indicator^23^.

### Hydrolysis procedure

**Figure 1:**
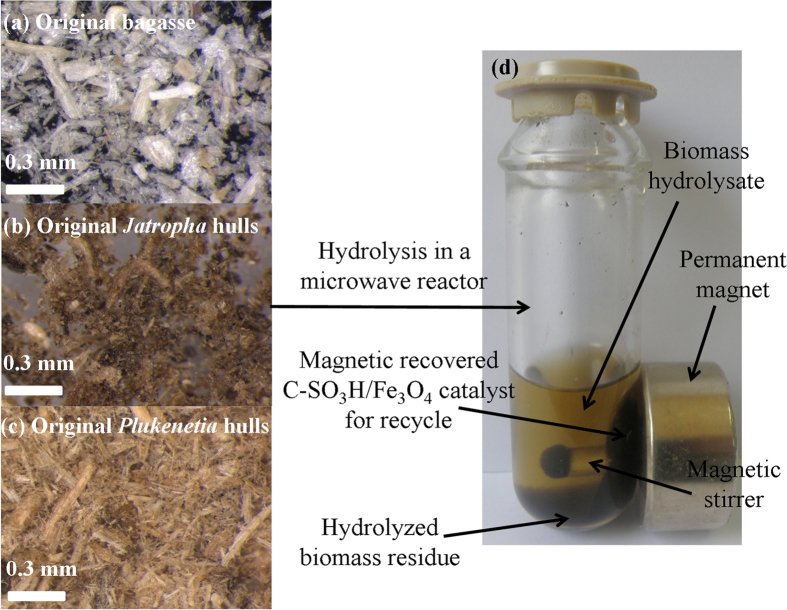
Photos of the tropical plant wastes and catalyst separation by a magnet after reaction.

Biomass (0.027 g), a certain amount of magnetic carbonaceous acid catalyst (0.06–0.27 g) and water (about 15 mL) were loaded in a 30 mL borosilicate vial for hydrolysis in a microwave reactor (Monowave 300, Anton Paar, Graz, Austria). The vial with samples was heated to 160–200 °C (0.5–2.2  MPa) in 3 min, and reacted for 2–4 h with magnetic stirring at 600 rpm. After reaction, catalyst was separated by a magnet, washed with deionized water and dried at 105 ^°^C for next cycles (Fig. 1). More details about the microwave experimental procedure can be seen in previous work^24^.

The optimized conditions for ball-milled cellulose by single-factor optimization were further applied to the hydrolysis of bagasse, *Jatropha* and *Plukenetia* hulls. All experiments were repeated twice with standard deviation (σ) of 0.02–3.26% for product yields and selectivities.

### Analyses of biomass wastes and hydrolysates

#### Compositions of biomass wastes

The components of the three tropical plant wastes were analyzed according to the technical report from US National Renewable Energy Laboratory (NREL)^25^. Water-ethanol extractives fraction (wt%) was determined by total weight loss after the extraction of biomass in refluxed water for 24 h and refluxed ethanol for another 24 h at their boiling points in a Soxhlet extractor. The extracted biomass was two-step acid hydrolyzed by 72% sulfuric acid stirred in a tube at 30 °C for 1 h and subsequent by 4% sulfuric acid at 121  °C for another 1 h in a sealed tube in an autoclave (HVE-50, Hirayama Manufacturing Corp., Tokyo), with a reference standard sugar mixture (glucose, xylose, arabinose and mannose) at similar concentrations of the sugars in biomass sample for calibration the decomposition of each sugar at the second step hydrolysis. Sugar (glucose, xylose, arabinose and mannose) fractions (wt%) in hydrolysate were analyzed by high-performance liquid chromatography (HPLC) described below and quantified using a blended calibration curve with six points for each standard (excluding zero) from 0.1 to 3 mg/mL (*R*^*2*^ = 0.9999). Acid-soluble lignin fraction (wt%) in the hydrolysate was measured by ultraviolet-visible (UV-Vis) spectrometer (UV-1800, Shimadzu, Kyoto) at 240 nm. Ash content (wt%) was determined by oxidation of the remaining solid residue in a muffle furnace (4–10, Yongguangming instrument factory, Beijing) at 575 °C for 24 h while acid-insoluble lignin fraction (wt%) was obtained by the difference of weight loss. The analysis was parallelly performed twice (Table 1).

#### Other characterized methods for biomass

XRD, SEM and FT-IR analyses were also performed for biomass similar for catalysts in above section. The degree of crystallinity (*CrI*) in biomass was calculated as the ratio of cellulose I in mixed crystalline structure from XRD data^26^:


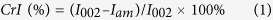


where *I*_*002*_ is the peak intensity at diffraction angle 2θ = 22°, whereas *I*_*am*_ is the height for the amorphous cellulose at 2θ = 18°.

#### Hydrolysate analyses

After hydrolysis, the aqueous solution was filtrated with a 0.22 μm filter for total organic carbon (TOC-VCPN; FA, CN200, Shimadzu), UV-Vis and HPLC (LC-20A, Shimadzu) analyses. In TOC analysis, standard glucose aqueous solution with four carbon concentrations (250, 500, 750 and 1000 mg/mL) was used to calibrate the curve (*R*^*2*^ = 0.9985). The concentration of total reducing sugars (TRS) in hydrolysate was determined using DNS (3,5-dinitrosalicylic acid) method with glucose as standard sample^27^. The absorbance of aqueous solution was measured at 540 nm using UV-Vis spectrometer, and TRS was quantified by external standard curve method with five glucose concentration (0.1, 0.2, 0.3, 0.4 and 0.5 mg/mL; *R*^*2*^ = 0.9987). Products (glucose, xylose, formic acid, acetic acid, HMF and furfural) in aqueous phase were also analyzed by HPLC equipped with an HPX-87H column, UV and refractive index detector, and calibrated with five standard points (0.1, 0.2, 0.4, 0.8 and 1.6 mg/mL, *R*^*2*^ = 0.9999). Sulfuric acid aqueous solution (0.01 mmol L^−1^) was used as mobile phase at flow rate of 0.6 mL min^−1^, and column temperature was 60 °C.

Product yields and selectivities (wt%) were defined as follows:


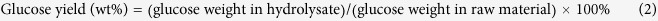



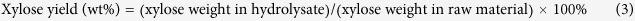



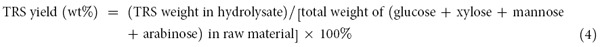



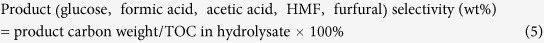


## Results and Discussion

**Figure 2:**
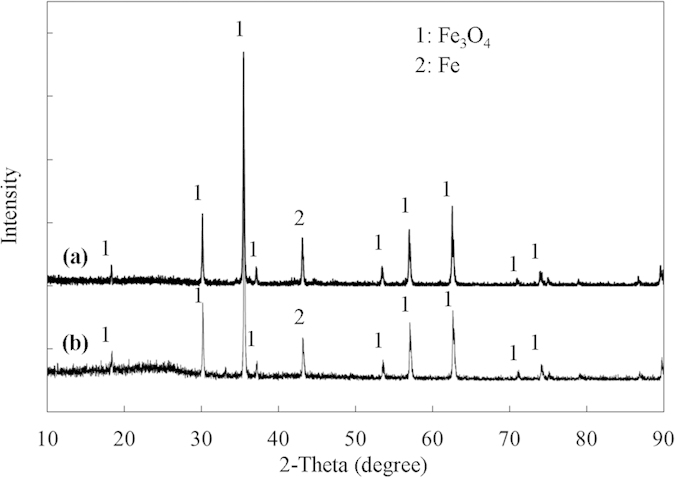
XRD patterns of the screened magnetic catalyst: (**a**) before sulfonation (C/Fe_3_O_4_) and (**b**) after sulfonation (C-SO_3_H/Fe_3_O_4_).

**Figure 3:**
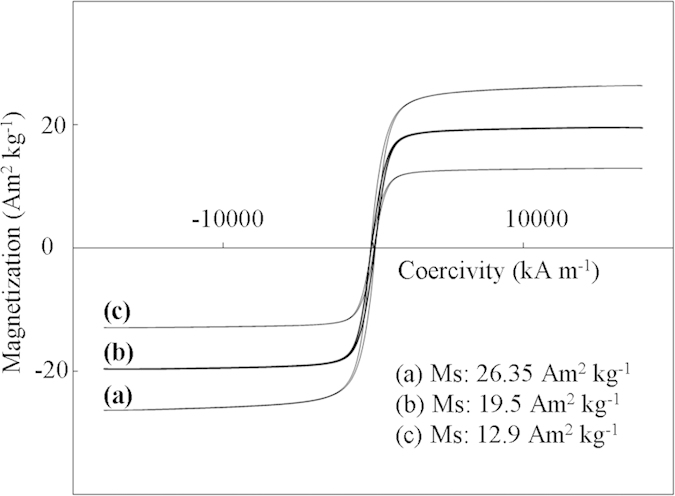
VSM curves for magnetic catalyst: (**a**) before sulfonation (C/Fe_3_O_4_), (**b**) after sulfonation (C-SO_3_H/Fe_3_O_4_), and (**c**) after seven cycles (C-SO_3_H/Fe_3_O_4_).

**Figure 4:**
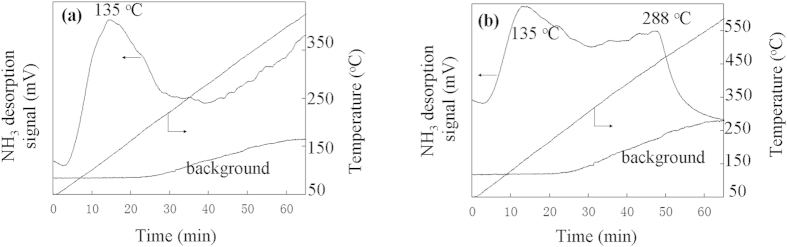
NH_3_-TPD curves of magnetic carbonaceous acid: (**a**) before sulfonation (C/Fe_3_O_4_, acidity of 0.17 mmol g^−1^) and (**b**) after sulfonation (C-SO_3_H/Fe_3_O_4_, acidity of 0.96 mmol g^−1^) (background without NH_3_).

**Figure 5:**
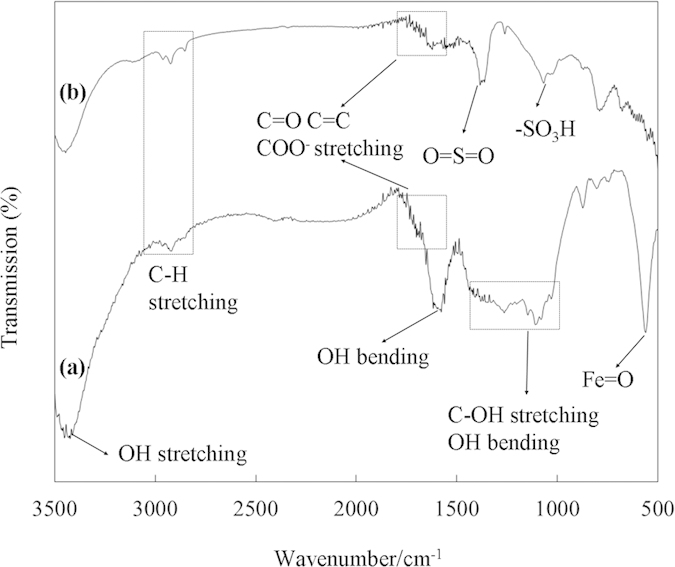
FT-IR spectra of magnetic catalyst: (a) before sulfonation (C/Fe_3_O_4_) and (b) after sulfonation (C-SO_3_H/Fe_3_O_4_).

**Figure 6:**
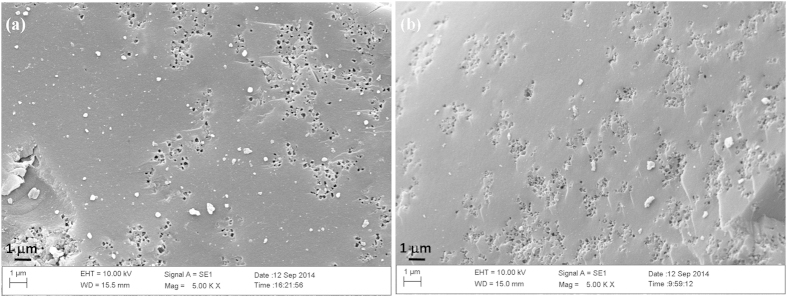
SEM images of magnetic catalyst: (**a**) before sulfonation (C/Fe_3_O_4_) and (**b**) after sulfonation (C-SO_3_H/Fe_3_O_4_).

**Figure 7:**
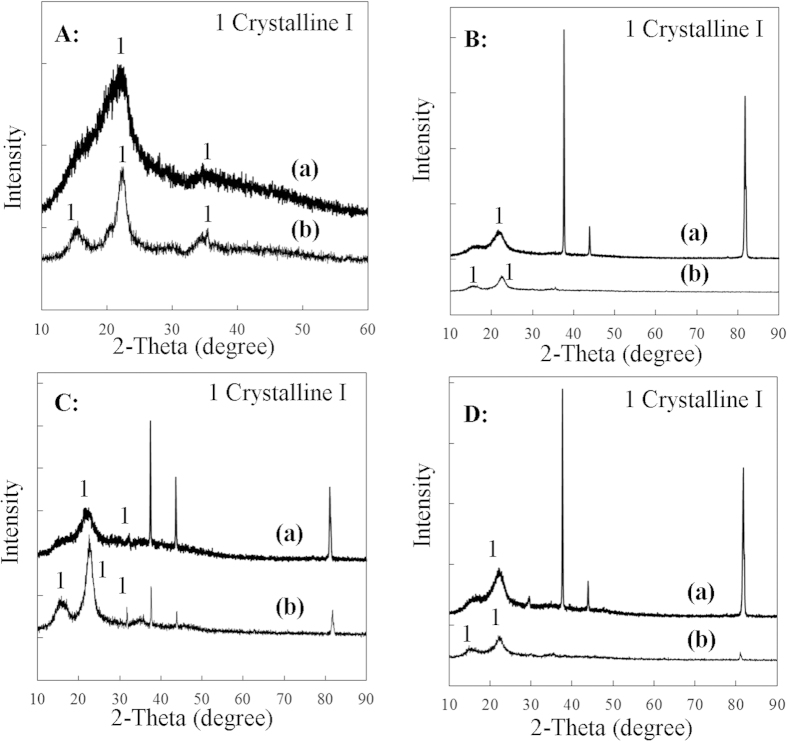
XRD patterns of original biomass materials: (**A**) ball-milled cellulose, (**B**) original bagasse, (**C**) original *Jatropha* hulls, and (**D**) original *Plukenetia* hulls (a: before reaction and b: after reaction).

**Figure 8:**
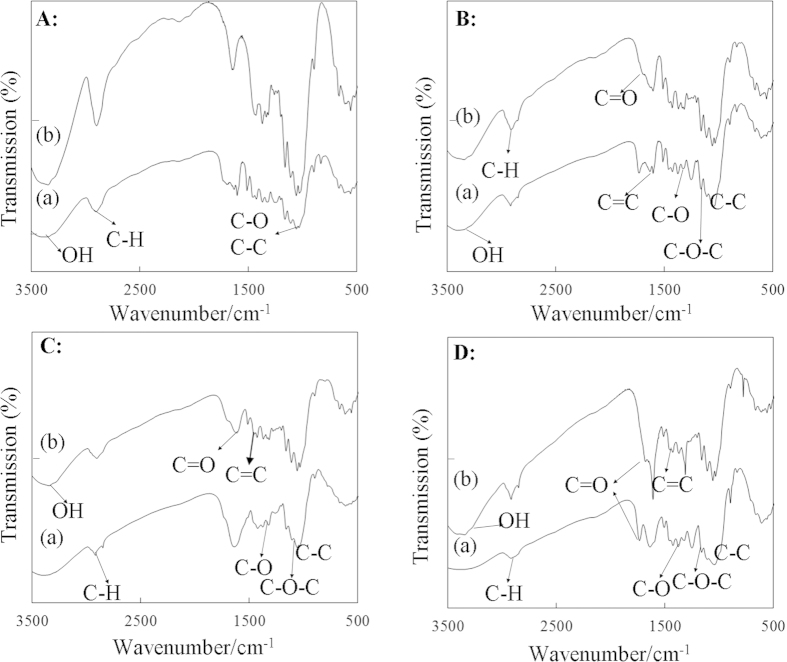
FT-IR spectra of biomass: (**A**) ball-milled cellulose, (**B**) original bagasse, (**D**) original *Jatropha* hulls, and (**D**) original *Plukenetia* hulls (a: before reaction and b: after reaction).

**Figure 9:**
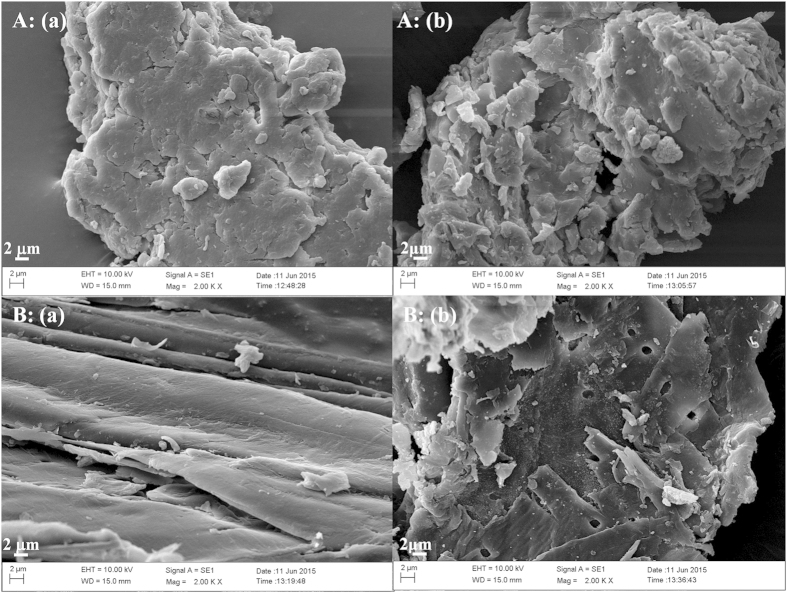
SEM images of (**A**) ball-milled cellulose and (**B**) original bagasse (a: before reaction and b: after reaction).

**Figure 10:**
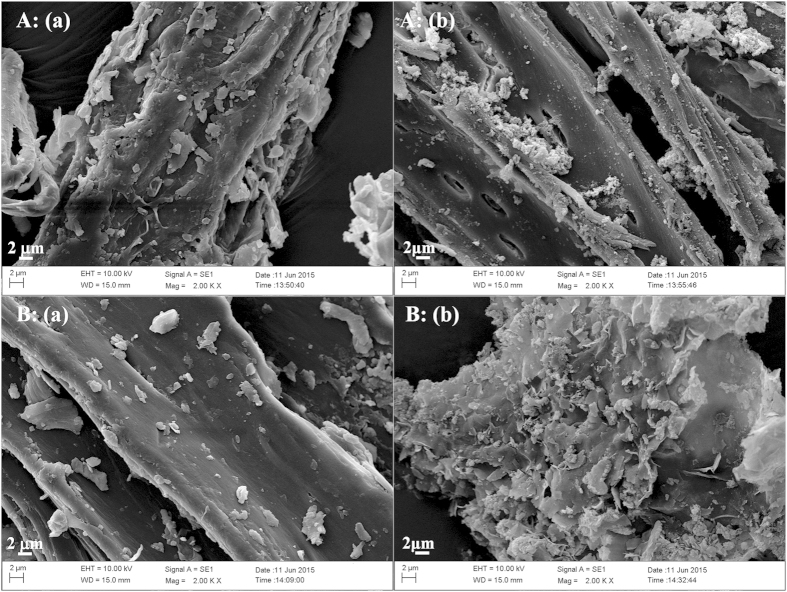
SEM images of (**A**) original *Jatropha* hulls and (**B**) original *Plukenetia* hulls (a: before reaction and b: after reaction).

**Figure 11:**
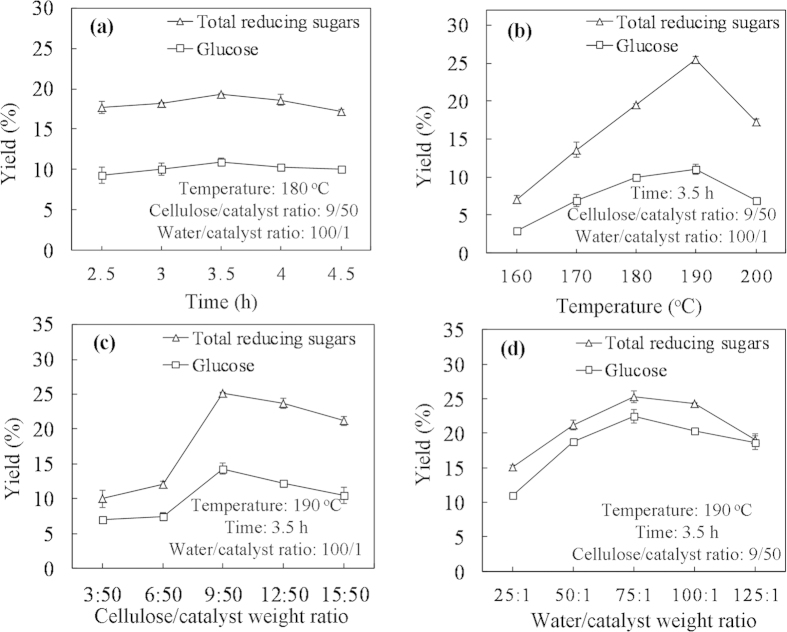
Single-factor test of ball-milled cellulose hydrolysis with C-SO_3_H/Fe_3_O_4_ catalyst: (**a**) time, (**b**) temperature, (**c**) cellulose/catalyst weight ratio, and (**d**) water/catalyst weight ratio.

**Figure 12:**
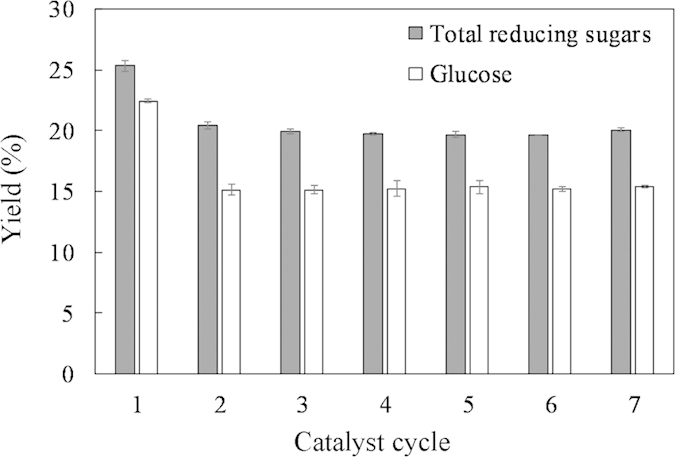
C-SO_3_H/Fe_3_O_4_ catalyst cycle for ball-milled cellulose hydrolysis.

Component analyses of the three tropical biomass wastes (*baga*sse, *Jatropha* and *Plukenetia* hulls) are summarized in Table 1. Magnetic catalysts were synthesized under different conditions according to an orthogonal design (Table 2), and their activities tested by cellulose hydrolysis are listed in Table 3. Photos for the tropical plant wastes and catalyst separation by a magnet after reaction are demonstrated in Fig. 1. XRD, VSM, NH_3_-TPD, FT-IR and SEM photos of the magnetic catalyst before and after sulfonation are presented in Figs 2, 3, 4, 5, 6, respectively. XRD, FT-IR and SEM photos of ball-milled cellulose, original tropical plant wastes before and after reaction are illustrated in Figs 7, 8, 9, 10, respectively. The screened magnetic catalyst is used for the single-factor optimization of cellulose hydrolysis with TRS and glucose yields illustrated in Fig. 11. Catalyst cycles for milled-cellulose hydrolysis is displayed in Fig. 12. The assignment of major IR absorptions of magnetic catalyst (before and after sulfonation) and biomass wastes (before and after reactions) was illustrated in Tables 4 and 5, respectively. The hydrolysis results for original, ball-milled, water-extracted, water and ethanol extracted tropical plant wastes are given in Table 6.

### Catalyst screening

Catalysts were prepared according to an orthogonal design [Table 2, L9(3^4^)] with four factors: weight ratio of glucose/Fe_3_O_4_ (6, 7.5, 9), carbonization temperature (650, 700, 750 °C), carbonization time (0.5, 1, 1.5 h) and sulfonation time (19, 20, 21 h). The optimized conditions for catalyst synthesis were glucose/Fe_3_O_4_ weight ratio of 6/1, carbonization temperature of 700 °C, carbonization time of 1 h and sulfonation time of 19 h, with the highest acid density of 0.40 mmol g^−1^ being achieved for the catalyst (Table 3; No# 10). In the presence of the optimized No# 10 catalyst (denoted as C-SO_3_H/Fe_3_O_4_), ball-milled cellulose was hydrolyzed at 180 °C for 3 h^28^, giving the highest TRS and glucose yields of 18.5% and 10.3%, respectively with hydrolysis yield [total organic carbon (TOC) in hydrolysate/carbon weight in cellulose × 100%] of 41.4%, that was slightly lower to the value (48.6%) over the magnetic catalyst (Fe_3_O_4_@C-SO_3_H) in previous work at 140 °C for 12 h^16^. The screened magnetic catalyst was further characterized and used for biomass hydrolysis below.

### Catalyst characterization

Figure 2 illustrated the crystalline structures of the catalyst before and after sulfonation (C/Fe_3_O_4_ *vs.* C-SO_3_H/Fe_3_O_4_). According to Joint Committee on Powder Diffraction Standard (JCPDS card No. 19–629), XRD peaks were identified as crystalline phases of Fe_3_O_4_ and Fe. Some of Fe_3_O_4_ was reduced to Fe by carbon in the pyrolysis process at 700 °C. There was no carbon peak detected, indicating carbon was in amorphous phase. During sulfonation, Fe_3_O_4_/Fe encapsulated in the carbon shell of catalyst converted little to iron sulphate by concentrated H_2_SO_4_, protected from the amorphous carbon^15^. However, some Fe_3_O_4_ particles remained on the surface after pyrolysis were converted to iron sulphate that was totally removed by washing several times with distilled water in the 50 mL autoclave at 200 °C for 3 h (detected using CaCl_2_). After sulfonation, no significant difference was seen in the crystalline phases between C/Fe_3_O_4_ and C-SO_3_H/Fe_3_O_4_.

Magnetic saturation (Ms) of the catalyst was 26.35, 19.5 and 12.9 Am^2^ kg^−1^ before sulfonation, after sulfonation, and after seven cycles for cellulose hydrolysis, respectively (Fig. 3). The decline in catalyst magnetism was due to the leaching of Fe_3_O_4_ by concentrated H_2_SO_4_ in sulfonation and residue deposition on the surface of catalyst during the cycles. The Ms value is comparable with that (23 Am^2^ kg^−1^) reported in literature^16^.

In Fig. 4, the catalyst (before and after sulfonation) showed a major desorption peak at 135 °C, because of the weak acid sites (-OH and -COOH) of incompletely formed carbon sheets^29^. After sulfonation, catalyst C-SO_3_H/Fe_3_O_4_ had a strong peak at 288 °C from −SO_3_H group with total acid density (mmol g^−1^) increased to 0.96 (*vs.* 0.17 for C/Fe_3_O_4_) as compared to 0.40 by elemental analysis and 0.75 by NaOH titration.

In Fig. 5, absorption at 566 cm^−1^ was from the Fe-O vibration in Fe_3_O_4_^30^, 1600–1800 cm^−1^ were from COO^−^, C=O and C=C stretching vibrations^31^, and 3463 cm^−1^ was from -OH stretching vibration with its hydrophilic surface for easier contact between reactant and catalyst^32^. After sulfonation, two bands at 1066 (-SO_3_^−^ stretching) and 1363 cm^−1^ (O=S=O stretching in -SO_3_H) appeared because the catalyst was functionalized with −SO_3_H group (Fig. 5b and Table 4).

SEM images (Fig. 6) showed the catalyst changed little after sulfonation, both particles had smooth surfaces with numerous pores on the surfaces.

### Biomass characterization

XRD results of four biomass materials before and after hydrolysis are given in Fig. 7. Ball-milled cellulose had a strong peak at angle (2θ) of about 22^o^, corresponding to crystalline cellulose I. the degree of crystallinity was only 41% calculated by equation (1). After hydrolysis, three crystalline peaks at angle (2θ) of about 16^o^, 22^o^, and 35^o^ became apparent, which were identified as the lattice plane (10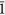), (002) and (040) of crystalline cellulose I, respectively^26^. So, the degree of crystallinity of cellulose after hydrolysis increased to 69%.The hydrogen-bonding structure of amorphous cellulose was much weaker than that of cellulose I, which made the amorphous part in ball-milled cellulose easier degraded in hydrolysis reaction, and left only crystalline cellulose I. Similar phenomenon was also observed for original bagasse, *Jatropha* and *Plukenetia* hulls (Fig. 7b–d) with the degree of cellulose crystallinity rose from 46%, 42% and 54% before hydrolysis to 78%, 72% and 60% after hydrolysis, respectively.

For ball-milled cellulose, absorptions at about 2880 and 3000–3500 cm^−1^ were due to C-H and -OH stretching, respectively, while absorption at 950–1200 cm^−1^ was from C-O/C-C stretching or C-H bending (Fig. 8 and Table 5)^33^. The original tropical plant wastes had an apparent characteristic absorption at 1155 cm^−1^ that was generally due to the C-O-C asymmetric bridge stretching of glycans, as well as characteristic absorptions at 1725 cm^−1^ for C=O stretching, 1599 and 1509 cm^−1^ for C=C stretching in aromatic ring and 1263 cm^−1^ related to C(O)-O stretching^34^. After hydrolysis, the main absorptions still existed, while the intensity of C-O stretching of glycans decreased, indicating the lignocellulosic structure remained although part of glycans were degraded (Fig. 8b–d).

The surface morphologies of ball-milled cellulose, original bagasse, *Jatropha* and *Plukenetia* hulls before and after reactions were observed by SEM (Figs 9 and 10). The four biomass samples showed a relatively compact and rigid surface before hydrolysis, but after reaction they exhibited a corrugated and more porous surface, revealing the disruption of tissue network and the destruction of highly ordered structure.

### Cellulose hydrolysis

#### Single-factor optimization

Referred to previous studies^12^,^35^, four factors including reaction time (2.5–4.5 h, excluding initial heating and final cooling times), reaction temperature (160–200 °C), cellulose/catalyst weight ratio (3/50–15/50) and water/catalyst weight ratio (25/1–125/1) were optimized for ball-milled cellulose hydrolysis by single-factor test, and the results were plotted in Fig. 11.

#### Reaction time

Under the fixed conditions (180 °C, cellulose/catalyst weight ratio of 9/50 and water/cellulose weight ratio of 100/1), the influence of reaction time was studied (Fig. 11a). Cellulose was first hydrolyzed to sugars and further degraded to small organic substances such as HMF, levulinic acid and formic acid^14^. As time increased from 2.5 to 3.5 h, TRS and glucose yields rose from 17.7% and 9.3% to 19.3% and 10.9%, respectively with minor byproducts levulinic acid (4.3%) and formic acid (2.1%), and slight HMF at 3.5 h. After 3.5 h, both TRS and glucose yields slightly decreased. Reaction time of 3.5 h was selected as the best value.

#### Reaction temperature

Reaction temperature from 160 to 200 °C was used to hydrolyze cellulose under the conditions (reaction time of 3.5 h, cellulose/catalyst weight ratio of 9/50 and water/cellulose weight ratio of 100/1) (Fig. 11b). At 160 °C, TRS (7.1%) and glucose yields (2.9%) were quite low. At 190 °C, the yields of TRS and glucose jumped sharply to 25.6% and 11.0%, respectively. However, at temperature >190 °C, they dropped remarkably owing to the decomposition to HMF and levulinic acid with the yields of 5.8% and 6.4%, respectively. The yields of levulinic acid and formic acid reached their high levels of 6.5% and 3.2%, respectively at 180 °C. Reaction temperature of 190 °C was selected for the following experiments.

#### Cellulose/catalyst weight ratio

Cellulose/catalyst weight ratio varied from 3/50 to 15/50 for cellulose hydrolysis with reaction time of 3.5 h, temperature of 190 °C and water/catalyst weight ratio of 100/1. In the absence of catalyst, only 3.4% TRS and 1.8% glucose yields were produced. In Fig. 11c, after catalyst (0.05 g) was added to the reaction system (cellulose/catalyst weight ratio of 3/50), 10.0% TRS and 7.0% glucose yields were obtained. The yields of TRS and glucose grew to 25.2% and 14.3%, respectively at higher cellulose/catalyst weight ratio of 9/50. However, as the weight ratio increased further from 9/50 to 15/50, TRS and glucose yields reduced slightly to 21.2% and 10.5%, respectively because of the enhancement of byproducts such as HMF (5.1%), acetic acid (3.2%) and formic acid (3.6%). So, cellulose/catalyst weight ratio of 9/50 was chosen as the best value. In previous literatures^11^,^12^,^16^, catalyst/cellulose weight ratio for cellulose hydrolysis was normally high (e.g., 1/1^11^, 12/1^12^ 3/1^16^ *vs.* 5.6/1 in this work) because of the weak acidity and heterogeneous reaction condition. However, considering the economic sustainability, the amount of solid catalyst should be limited. Decreasing the crystallinity of cellulosic materials by pretreatment, improving the activity of solid catalysts and enhancing the mixture of reactants (e.g., by ultrasound) may be the possible methods to achieve the purpose.

#### Water/catalyst weight ratio

Water/catalyst weight ratio was changed from 25/1 to 125/1 under the conditions of reaction time of 3.5 h, temperature of 190 °C and cellulose/catalyst weight ratio of 9/50 for cellulose hydrolysis (Fig. 11d). TRS and glucose yields increased from 15.1% and 11.0% to 25.3% and 22.4%, respectively as water/catalyst weight ratio grew from 25/1 to 75/1, meanwhile, glucose selectivity reached 45.0%. As the weight ratio rose further from 75/1 to 125/1, TRS and glucose yields declined to 19.1% and 18.7%, respectively because of the leaching or deactivation of the active sites of catalyst by excess water^30^. Therefore, the best value for water/catalyst weight ratio was 75/1.

In conclusion, the selected best conditions for ball-milled cellulose hydrolysis with glucose yield of 22.4%, TRS yield of 25.3% and glucose selectivity of 48% were as follows: reaction time of 3.5 h, reaction temperature of 190 °C, cellulose/catalyst weight ratio of 9/50 and water/catalyst weight ratio of 75/1. The above conditions were used in the following experiments for catalyst cycles and hydrolysis of the tropical biomass wastes.

### Catalyst cycles

Under the above best conditions, C-SO_3_H/Fe_3_O_4_ catalyst was conducted recycle experiments for ball-milled cellulose hydrolysis (Fig. 12). The recovery rate of catalyst was 92.8% after seven cycles. In the second cycle, TRS and glucose yields decreased from 25.3% and 22.4% to 20.5% and 15.1%, respectively. Correspondingly, the total acid density of the catalyst decreased from 0.96 to 0.83 mmol g^−1^ after one cycle by NH_3_-TPD analysis. However, both TRS and glucose yields changed little after the second cycle (20.1% TRS and 15.4% glucose yields at the seventh cycle), indicating good stability of the second cycled catalyst. NH_3_-TPD analysis also showed the relative stable acid density (0.83 and 0.70 mmol g^−1^ after the first and seventh cycles). On the other hand, the total acid density was 0.75 for fresh catalyst and 0.63 mmol g^−1^ after seven cycles by NaOH titration.

In conclusion, C-SO_3_H/Fe_3_O_4_ catalyst can cycle at least seven times with relatively high TRS yield of 20.1%, glucose yield of 15.4% and catalyst recovery of 92.8% at the seventh cycle.

### Hydrolysis of tropical biomass wastes

Under the best conditions obtained for cellulose, the catalyst was applied to the hydrolysis of original, ball-milled, water-extracted, water and ethanol extracted tropical plant wastes: bagasse, *Jatropha* and *Plukenetia* hulls (Table 6). Every experiment was conducted in duplicate.

For original bagasse hydrolysis, 57.0% TRS, 42.6% glucose and 95.7% xylose yields were obtained at 190 °C for 3.5 h that were much higher than those yields from ball-milled cellulose. It demonstrated that the interpolation and bonding of hemicellulose with cellulose in native plants destroyed the rigorous and recalcitrant structure of pure cellulose to benefit the degradation of cellulose part^4^. However, the degradation of monosaccharides to byproducts such as acetic acid, furfural and HMF was also promoted with the yields of 6.0%, 5.6% and 3.1%, respectively.

Compared with bagasse, the glucan and xylan contents of *Jatropha* and *Plukenetia* hulls were lower (34.1% and 30.5% *vs.* 40.1%; 10.3% and 15.1% *vs.* 18.5%, Table 1). The remarkable difference of *Jatropha* and *Plukenetia* hulls from bagasse was that the hulls contain higher extractives (16.8% and 17.6% *vs.* 9.8%), such as fatty oils and phenols (identified before for *Jatropha* hulls^36^). These extractives cannot be hydrolyzed and bonded to glucan to prevent it contact with water molecules and catalyst for hydrolysis. *Jatropha* and *Plukenetia* hulls had low TRS yields of 29.5% and 34.0% (*vs.* 57.0% for bagasse), respectively. Glucose yield was also reduced sharply from 42.6% for bagasse to 22.2% and 20.5% for *Jatropha* and *Plukenetia* hulls, while xylose yield still kept high with little change due to the easy hydrolysis for xylan.

Pretreatments of the tropical biomass samples by ball-milling, water extraction, water-ethanol extraction were conducted to destroy biomass structure or remove extractives to improve the production of TRS and glucose. After ball-milled, TRS and glucose yields of 68.4% and 51.1% for bagasse, 35.4% and 26.7% for *Jatropha* hulls, as well as 40.8% and 24.6% for *Plukenetia* hulls were obtained, which was attributed to easier hydrolysis because of the structural damage of crystalline cellulose and extractives. For water-ethanol extracted bagasse, *Jatropha* and *Plukenetia* hulls, TRS and glucose yields increased to 79.8% and 58.3% (*vs.* 57% and 42.6%), 47.2% and 35.6% (*vs.* 29.5% and 22.2%), 54.4% and 35.8% (*vs.* 34% and 20.5%), respectively compared with original materials, which could be due to the removal of extractable composition in raw biomass that hindered biomass hydrolysis and catalyst activity. Meanwhile, the formation of by-products such as furfurals and small organic acids in biomass hydrolysis had no significant changes, which made the product selectivity of glucose from cellulose remarkably increased after pretreatment.

It can be concluded that the catalyst showed a better activity in the hydrolysis of bagasse. For original bagasse, *Jatropha* and *Plukenetia* hulls, proper treatment such as ball-milling and solvent extraction improved hydrolysis yields significantly.

## Conclusions

Catalyst C-SO_3_H/Fe_3_O_4_ with strong magnetism (19.5 Am^2^ kg^−1^) and high total acid density (0.75 mmol g^−1^, NaOH titration) was synthesized and screened for the hydrolysis of cellulose and tropical plant wastes in microwave reactor. It was cycled seven times with little deactivation and magnetism loss. The best hydrolysis conditions for ball-milled cellulose were optimized with single-factor optimization with the yields of reducing sugars and glucose of 25.3% and 22.4%, respectively. The catalyst was used to hydrolyze bagasse, *Jatropha* and *Plukenetia* hulls with the highest glucose yield of 58.3%, 35.6% and 35.8%, respectively. Pretreatment of tropical biomass greatly improved hydrolysis yield and glucose selectivity.

## Additional Information

**How to cite this article**: Su, T.-C. *et al.* Hydrolysis of Selected Tropical Plant Wastes Catalyzed by a Magnetic Carbonaceous Acid with Microwave. *Sci. Rep.* **5**, 17538; doi: 10.1038/srep17538 (2015).

## Figures and Tables

**Table 1:** Components of tropical biomass wastes (wt%)^a^.

Biomass	Glucan (%)	Xylan (%)	Arabian (%)	Mannosan (%)	Lignin (%)	Ash (%)	Extractives (%)
Bagasse	40.1 ± 2.1	18.5 ± 0.8	4.4 ± 2.3	3.2 ± 0.8	20.7 ± 3.1	3.7 ± 0.9	9.8 ± 3.1
*Jatropha* hulls	34.1 ± 1.8	10.3 ± 1.5	3.7 ± 1.3	1.0 ± 0.6	18.4 ± 2.0	3.2 ± 0.8	16.8 ± 0.6
*Plukenetia* hulls	30.5 ± 1.6	15.1 ± 0.5	2.7 ± 1.9	1.2 ± 2.5	17.3 ± 1.7	4.7 ± 2.7	17.6 ± 1.8

^a^By NRNL method^25^ analyzed with 2 repeats.

**Table 2:** Orthogonal design for catalysts synthesized under different conditions.

Catalyst number	Weight ratio of glucose/Fe_3_O_4_	Carbonization temperature (°C)	Carbonization time (h)	Sulfonation time (h) at 150 ^°^C
1	6	650	0.5	19
2	6	700	1	20
3	6	750	1.5	21
4	7.5	650	1	21
5	7.5	700	1.5	19
6	7.5	750	0.5	20
7	9	650	1.5	20
8	9	700	0.5	21
9	9	750	1	19
10	6	700	1	19

**Table 3:** Activity of catalysts synthesized under different conditions for ball-milled cellulose hydrolysis.

Catalyst number	N (%)	C (%)	H (%)	S (%)	Acid density by S content (mmol g^−1^)	TRS yield (%)^a^	Glucose yield (%)^a^	Glucose selectivity (%)^a^
1	0.061	55.50	2.00	1.15	0.36	17.1 ± 1.4	9.1 ± 2.4	39.6 ± 2.4
2	0.052	58.00	1.82	1.02	0.32	17.7 ± 1.6	10.2 ± 0.4	41.6 ± 2.8
3	0.058	53.87	1.99	1.11	0.35	17.1 ± 0.5	8.0 ± 2.1	37.5 ± 1.6
4	0.061	55.50	2.00	1.15	0.36	17.2 ± 0.5	9.6 ± 2.3	41.8 ± 2.8
5	0.066	56.53	2.04	1.08	0.34	17.5 ± 1.7	9.2 ± 1.9	39.5 ± 1.5
6	0.039	60.91	2.01	0.91	0.28	16.9 ± 1.9	8.9 ± 0.4	40.5 ± 2.5
7	0.063	59.98	2.17	1.00	0.31	14.8 ± 2.4	7.2 ± 0.7	36.2 ± 0.3
8	0.060	61.79	2.10	0.97	0.30	17.7 ± 3.1	8.1 ± 0.8	36.8 ± 1.9
9	0.060	61.62	2.13	0.95	0.30	13.5 ± 2.9	6.1 ± 1.3	34.3 ± 0.8
10	0.034	53.00	2.10	1.28	0.40	18.5 ± 1.5	10.3 ± 2.1	41.4 ± 3.2

^a^Conditions for hydrolysis reactions: ball-milled cellulose 0.027 g, catalyst 0.15 g, water 15 mL, 180 °C for 3 h. Two repetitions were done for each run.

**Table 4:** Major IR absorptions of magnetic catalyst before and after sulfonation (Fig. 5)^30^,^31^,^32^.

Major absorptions	Wavenumber (cm^−1^)	
	C/Fe_3_O_4_ (Fig. 5a)	C-SO_3_H/Fe_3_O_4_ (Fig. 5b)
Fe=O stretching	566	538
−SO_3_^-^ stretching	−	1066
O=S=O stretching	−	1363
OH bending	1686	1636
C=O, C=C, COO^−^ stretching	1600–1800	1600–1800
C-H stretching	2863–3075	2863–3075
OH stretching	3463	3438

**Table 5:** Major IR absorptions of biomass wastes before and after reactions (Fig. 8)^33^,^34^.

Major absorptions	Wavenumber (cm^−1^)							
	Cellulose (Fig. 8A)		Bagasse (Fig. 8B)		*Jatropha* hulls (Fig. 8C)		*Jatropha* hulls (Fig. 8D)	
	Before hydrolysis	After hydrolysis	Before hydrolysis	After hydrolysis	Before hydrolysis	After hydrolysis	Before hydrolysis	After hydrolysis
C-C stretching	1036	1045	950–1200	950–1200	950–1200	950–1200	950–1200	950–1200
C-O-C stretching	–	–	1155	1128	1086	1032	1123	1076
C-O stretching	1256	1260	1263	1203	1203	1163	1236	1176
C=C stretching	–	–	1599	1518	1508	1558	1575	1535
C=O stretching	–	–	1725	1683	1686	1657	1701	1635
C-H stretching	2882	2878	2880	2826	2763	2704	2781	2808
-OH stretching	3463	3446	3463	3369	3456	3218	3438	3357

**Table 6:** Hydrolysis of the three tropical biomass wastes^a^.

Biomass	Yield (%)			Product selectivity (%)				
	TRS	Glucose	Xylose	Glucose	Formic acid	Acetic acid	HMF	Furfural
Bagasse								
original	57.0 ± 1.8	42.6 ± 3.5	95.7 ± 1.9	60.1 ± 1.4	1.2 ± 0.9	6.0 ± 1.6	3.1 ± 0.3	5.6 ± 1.2
ball-milled	68.4 ± 1.2	51.1 ± 1.4	96.1 ± 1.2	71.1 ± 0.7	1.8 ± 0.5	4.7 ± 3.1	5.2 ± 2.4	6.3 ± 2.5
water-extracted	71.3 ± 0.9	54.1 ± 3.1	94.7 ± 2.1	77.4 ± 1.7	1.9 ± 1.2	5.1 ± 1.6	4.9 ± 1.4	5.8 ± 2.7
water-ethanol extracted	79.8 ± 1.2	58.3 ± 2.1	97.2 ± 1.6	82.2 ± 3.1	2.3 ± 3.2	4.6 ± 3.2	5.0 ± 3.0	4.9 ± 1.6
*Jatropha* hulls								
original	29.5 ± 2.1	22.2 ± 2.5	95.3 ± 3.2	46.7 ± 2.0	6.9 ± 1.6	2.7 ± 0.8	0.2 ± 0.2	0.0
ball-milled	35.4 ± 3.1	26.7 ± 0.4	95.6 ± 2.1	56.0 ± 3.1	6.2 ± 0.4	2.4 ± 1.4	1.4 ± 0.2	0.8 ± 1.8
water-extracted	41.2 ± 1.5	31.1 ± 1.3	94.8 ± 1.0	63.4 ± 1.5	5.9 ± 1.1	2.6 ± 1.1	1.6 ± 1.1	1.2 ± 0.7
water-ethanol extracted	47.2 ± 0.6	35.6 ± 1.9	96.4 ± 2.6	70.1 ± 0.4	6.0 ± 0.9	3.1 ± 0.6	2.3 ± 0.1	0.9 ± 0.2
*Plukenetia* hulls								
original	34.0 ± 2.2	20.5 ± 1.6	97.9 ± 2.6	42.8 ± 0.7	5.1 ± 0.8	8.3 ± 1.1	0.5 ± 0.4	3.1 ± 0.7
ball-milled	40.8 ± 1.4	24.6 ± 1.2	96.8 ± 0.3	50.4 ± 3.0	4.9 ± 2.5	7.6 ± 1.7	1.3 ± 0.9	3.4 ± 1.7
water-extracted	44.2 ± 1.2	28.7 ± 0.6	95.7 ± 1.3	58.9 ± 0.3	3.8 ± 1.1	6.2 ± 0.7	1.5 ± 1.3	2.8 ± 1.1
water-ethanol extracted	54.4 ± 0.4	35.8 ± 1.3	94.9 ± 0.9	68.5 ± 1.5	4.5 ± 0.5	7.4 ± 0.7	1.5 ± 1.1	3.5 ± 0.9

^a^Conditions for hydrolysis reactions: biomass 0.027 g, catalyst 0.15 g, water 11.25 mL, 190 °C for 3.5 h. Two repetitions were done for each run.
